# Matrix metalloproteinase protein expression profiles cannot distinguish between normal and early osteoarthritic synovial fluid

**DOI:** 10.1186/1471-2474-13-126

**Published:** 2012-07-23

**Authors:** Bryan J Heard, Liam Martin, Jerome B Rattner, Cyril B Frank, David A Hart, Roman Krawetz

**Affiliations:** 1 Faculty of Medicine, University of Calgary, Calgary, AB, Canada; 2 Department of Cell Biology and Anatomy, University of Calgary, Calgary, AB, Canada; 3 Department of Surgery, University of Calgary, Calgary, AB, Canada; 4 McCaig Institute for Bone & Joint Health, Faculty of Medicine, University of Calgary, 3330 Hospital Drive NW, T2N 4N1, Calgary, AB, Canada

## Abstract

**Background:**

Osteoarthritis (OA) and Rheumatoid arthritis (RA) are diseases which result in the degeneration of the joint surface articular cartilage. Matrix Metalloproteinases (MMPs) are enzymes that aid in the natural remodelling of tissues throughout the body including cartilage. However, some MMPs have been implicated in the progression of OA and RA as their expression levels and activation states can change dramatically with the onset of disease. Yet, it remains unknown if normal and arthritic joints demonstrate unique MMPs expression profiles, and if so, can the MMP expression profile be used to identify patients with early OA. In this study, the synovial fluid protein expression levels for MMPs 1, 2, 3, 7, 8, 9, 12 & 13, as well as those for the Tissue Inhibitors of MMPs (TIMPs) 1, 2, 3, & 4 were examined in highly characterized normal knee joints, and knee joints with clinically diagnosed OA (early and advanced) or RA. The purpose of this study was to determine if normal, OA, and RA patients exhibit unique expression profiles for a sub-set of MMPs, and if early OA patients have a unique MMP expression profile that could be used as an early diagnostic marker.

**Methods:**

Synovial fluid was aspirated from stringently characterized normal knee joints, and in joints diagnosed with either OA (early and advanced) or RA. Multiplexing technology was employed to quantify protein expression levels for 8 MMPs and 4 TIMPs in the synovial fluid of 12 patients with early OA, 17 patients diagnosed with advanced OA, 15 with RA and 25 normal knee joints. Principle component analysis (PCA) was used to reveal which MMPs were most influential in the distinction between treatment groups. K – means clustering was used to verify the visual grouping of subjects via PCA.

**Results:**

Significant differences in the expression levels of MMPs and TIMPs were observed between normal and arthritic synovial fluids (with the exception of MMP 12). PCA demonstrated that MMPs 2, 8 & 9 can be used to effectively separate individuals diagnosed with advanced arthritis from early osteoarthritic and normal individuals, however, these MMP profiles do not separate early OA from normal synovial fluid. An apparent separation between advanced OA and RA subjects was also revealed through PCA. K-means clustering verified the presence of 3 clusters: normal joints clustered with early OA, and separate clusters of advanced OA or RA.

**Conclusions:**

This study demonstrates that unique MMP and TIMP expression profiles are present within normal, advanced OA and RA synovial fluid. These MMP profiles can be used to distinguish advanced OA & RA synovial fluid from early OA & normal synovial fluid, and even between synovial fluid samples from OA and RA joints. Although this methodology cannot be used for the diagnosis of early OA, high throughput multiplex technology of MMPs and TIMPs in synovial fluid may prove useful in determining the severity of the disease state, and/or quantifying the response of individuals to disease interventions.

## Background

There are likely several clinically recognized arthritic phenotypes, however, broadly speaking there are two main ‘types’ of arthritis: RA, a chronic inflammatory disease characterized by joint swelling, joint tenderness, and destruction of synovial joints [1] and OA, a heterogeneous group of conditions that lead to joint symptoms and signs which are associated with the defective integrity of articular cartilage [2]. Given the presence of auto-antibodies to a number of proteins such as citrullinated peptides (CP) and immunoglobulin (i.e. rheumatoid factor: RF) [1], RA has been appropriately classified as an autoimmune disease. There are a number of validated diagnostic tools and criteria that can predict the onset of RA, in some cases years before overt clinical manifestations. However, in the case of OA, no known (validated) early diagnostic test exists that can either predict the onset or eventual severity of the disease. To this end, a number of studies have used structural fragments of cartilage proteins as potential biomarkers of OA. Procollagen II C-propeptide upregulation, a precursor of COL II that appears as a repair response to damaged cartilage, has been localized in the articular cartilage of patients diagnosed with early OA [3]. Studies have shown this up-regulation is detectable in patient synovial fluid and serum, thus highlighting it as a potential biomarker for early OA [3]. Conversely, proteolytic COL II breakdown is key in the cartilage erosion witnessed in arthritic diseases. Fragments of COL II or COL II epitopes have also been highlighted as potential OA biomarkers, generally falling into one of three potential categories based on their origin from the native COL II molecule: cleavage neoepitopes, denaturation epitopes, or epitopes from the mature end of the molecule [4]. To date, however, no COL II/ProCOL II early OA diagnostic assays have entered the market due to the variability of COL II epitopes within normal and arthritic patient populations. Other historical biomarkers such as cartilage oligomeric protein (COMP) and hyaluronan (HA) [5,6] have also been identified as early biomarkers, but have not been clinically adopted. More recently, profiling of the inflammatory response in OA patients is being investigated, however, this area of study is still in need of further investigation [7,8]. Clinically speaking, only when evidence of joint space narrowing, cartilage loss, or osteophyte formation is obtained can the disease be clinically diagnosed in its later stages [2]. In this capacity, matrix metalloproteinases (MMPs) have been studied as potential indicators of the onset of arthritis as MMPs have been implicated in the loss of articular cartilage which occurs both in OA and RA [9,10].

The chondrocyte extracellular matrix (ECM) of healthy and arthritic joints is constantly subjected to remodelling processes [11,12]. This delicate balance between anabolism and catabolism of ECM components is mediated by interactions with synthetic and degradative molecules present in the joint environment (synovial fluid). In arthritic conditions, it is hypothesised that for some reason the catabolic potential within the joint environment overrides the anabolic, resulting in irreversible destruction within the joint. Many proteolytic enzymes have the ability to degrade the articular cartilage collagen scaffold including several of the MMPs [13,14], as well as non-MMPs. MMPs are a family of tissue remodeling enzymes which, when activated, recognize specific sequences in ECM proteins and then cleave these proteins. MMP-1 is an interstitial collagenase (collagenase 1) produced mainly (but not exclusively) by synovial cells within the joint. MMP-1 efficiently degrades collagen types I and III during fibrolytic tissue remodeling, while also maintaining the ability to degrade type II collagen. MMP-2 is a gelatinase (gelatinase A) that is secreted by stromal cells beneath the synovial lining [15]. This MMP has the ability to degrade aggrecan, and extracellular matrix proteoglycan, as well as collagen types I, II, and III using a similar mechanism to MMPs of the collagenase subfamily. MMP-3 is a stromelysin (stromelysin 1) with a specificity to facilitate degradation of extracellular matrix proteoglycans such as versican and aggrecan. MMP-7, matrilysin, has been show to cleave proteoglycans and collagen III/IV/V/IX/X/XI when present in its active form [16]. MMP 7 in its active form has also been shown to activate pro-MMPs 1, 2, and 7 [16]. MMP-8, neutrophil collagenase, is secreted by neutrophils and MMP-9, gelatinase-B, is also secreted by neutrophils in addition to macrophages and synovial cells [15]. MMP-12, a metalloelastase secreted by macrophages, has the ability to breakdown collagen type IV, and proteoglycan, as well as various other ECM components [17]. MMP-13 is a collagenase (collagenase 3) produced by chondrocytes and has specificity for type II collagen, while to a lesser extent also degrades collagen types I and III as well as aggrecan. MMPs are inhibited by specific endogenous tissue inhibitors of metalloproteinases (TIMPs), which consist of four protease inhibitors: TIMP1, TIMP2, TIMP3 and TIMP4. Each TIMP binds with differential rates of interaction and affinity to a MMP, (1:1, 2:2 stoichiometry). TIMP-1 inhibits MMP-1, MMP-3 and MMP-9 [18-21]. TIMP-2 inhibits proMMP-2 [18,22], however, TIMP-2 also has a bi-functional effect on MMP-2 since proMMP-2 activation requires low levels of TIMP-2 for activation, whereas a greater concentration of TIMP-2 inhibits MMP-2 [23]. TIMP-3 inhibits MMP-2 and MMP-9 [24], whereas TIMP-4 is a good inhibitor for all classes of MMPs without remarkable preference for specific MMPs [25].

In this study, the concentrations of specific MMPs (1, 2, 3, 7, 8, 9, 12, and 13) were quantified in the synovial fluid collected from normal joints and joints from patients clinically diagnosed with OA (early and advanced) or RA. Through PCA and K – means clustering analysis, reproducible and unique MMP expressions profiles were identified for normal, OA (advanced) and RA synovial fluid, however, normal and early OA synovial fluid cannot be distinguished from each other using this methodology. Utilizing high-through-put multiplexing methodologies with multi-variant statistical analysis allows for the visualization of changes within a certain marker between/within groups, but also provides the opportunity to observe if the interaction of many markers can better describe a phenotype of arthritic disease. This is of significance as distinct MMP profiles of disease may be exploited for alternative diagnostics as well as tracking disease activity/severity with therapeutic intervention.

## Methods

### Ethics Statement

Informed consent to participate was obtained by written agreement. The study protocol was approved by the University of Calgary Research Ethics Board (Application number: 21987).

#### Inclusion and Exclusion Criteria

Early OA Group: Inclusion criteria was early OA diagnosed based on arthroscopic examination, these patients had an Outerbridge score of under grade 2 (a partial-thickness defect with fissures on the surface that do not reach sub-chondral bone or exceed 1.5 cm in diameter), all but one patient was over 40 years of age. Advanced OA Group: Inclusion criteria were an age of 40 years or older, OA diagnosed based on the American College of Rheumatology [2] criteria with X-ray documentation, and no evidence of autoimmune disease or RA.RA Group: Inclusion criteria were an age of 40 years or older, RA diagnosed based on the American College of Rheumatology criteria [1].

Normal Group: Inclusion criteria for control cadaveric donations (collected at the Southern Alberta Organ and Tissue Donation Program (SAOTDP)) were an age of 40 years or older, no history of arthritis, joint injury or surgery (including visual inspection of the cartilage surfaces during recovery), no prescription anti-inflammatory medications, no co-morbidities (such as diabetes/cancer), and availability within 4 hrs of death.

### Subjects

Early OA Group: Twelve patients (11 males, ages: 31, 43, 46, 46, 49, 50, 52, 54, 58, 59, 66 / 1 female, age: 49) diagnosed with early osteoarthritis had synovial fluid aspirated prior to the arthroscopic examination without lavage (Table 1) (University of Calgary Ethics # 21987).

**Table 1:** Summary of normal, early OA, OA, and RA individuals

**Patient Group**	**Age**	**Sex**	**Clinical Diagnosis**	**Prescribed Medication**
Early Osteoarthritis (n=12)	58	M	Left knee	N/A
	43	M	right knee	N/A
	43	M	right knee	N/A
	49	F	right knee	N/A
	49	M	right knee	N/A
	66	M	right knee	N/A
	54	M	Left knee	N/A
	59	M	Left knee	N/A
	50	M	right knee	N/A
	46	M	right knee	N/A
	46	M	right knee	N/A
	31	M	right knee	N/A
	52	M	right knee	N/A
Osteoarthritis (n=17)	51	M	OA Left knee	N/A
	57	M	OA Left knee	N/A
	62	M	OA Left knee	N/A
	68	M	OA Both Knees & Spine	N/A
	75	M	OA Both Knees & Spine	N/A
	52	F	OA Right knee	N/A
	55	F	OA Right knee	N/A
	64	F	OA Left knee	N/A
	73	F	OA Left knee	N/A
	74	F	OA Both Knees	N/A
	65	M	OA Both Knees	N/A
	68	M	Knee & Both Hands	N/A
	62	F	Left Knees	N/A
	58	F	Both Knees & Spine	N/A
	49	F	Right Knee	N/A
	43	F	Both Knees	N/A
	45	M	Right Knee	N/A
Rheumatoid Arthritis (n=15)	40	M	RA	Infiximab
	51	M	RA	Leftlunomide
	55	M	RA	N/A
	40	F	RA	Methotrexate, Plaquenil, Abatacept
	48	M	RA	N/A
	49	F	RA	Methotrexate
	54	F	RA	Abatacept
	61	F	RA	N/A
	75	F	RA	Hydrochloroquine, Naproxen
	75	M	RA	Prednisone
	52	M	RA	N/A
	54	M	RA	Methotrexate
	48	M	RA	Abatacept
	63	M	RA	Abatacept
	55	M	RA	Methotrexate

Normal (n=25) 58±11 14M, 11F Normal Joint – No prescribed medications.

Synovial fluid was aspirated from the knees of normal individuals and patients with OA or RA. The age, sex, afflicted joint(s) and prescribed medication are presented above.

Advanced OA Group: Seventeen patients (8 males, ages: 45, 51, 57, 62, 65, 68, 68, 75 / 9 females, ages: 43, 49, 52, 55, 58, 62, 64, 73, 74) diagnosed with osteoarthritis had synovial fluid aspirated during routine medical visits without lavage (Table 1) (University of Calgary Ethics # 21987).

RA Group: Fifteen patients (5 males, ages: 40, 48, 51, 55, 55 / 10 females, ages: 40, 48, 49, 52, 54, 54, 61, 63, 75) diagnosed with Rheumatoid arthritis had synovial fluid aspirated during routine medical visits without lavage (Table 1)( University of Calgary Ethics # 21987).

Normal Control Group: Thirty fresh cadaveric donations were recovered within 4 hours from time of death through the SAOTDP (University of Calgary Ethics # 21987). Synovial fluid was collected from the remaining 25 normal individuals without lavage (14 males, ages: 40, 47, 50, 52, 54, 54, 56, 58, 65, 65, 68, 69, 72, 77 / 11 females, ages: 42, 46, 48, 50, 51, 51, 52, 65, 65, 68, 75, 78) (Table 1).

### Synovial Fluid

Synovial fluid from control individuals was obtained by the SAOTDP. Synovial fluid from advanced OA and RA patients was aspirated from the knee joint by the attending Rheumatologist using conventional sterile technique. The early OA synovial fluid was recovered by an orthopedic surgeon under sterile conditions. All synovial fluid samples were collected without the use of lavage or any other diluant. The native synovial fluid samples were aliquoted and stored in cryogenic vials at -80^0^ C after cells were removed by centrifugation at 4^0^ C. For standardization of the protocol, all synovial fluid samples were subjected to only one freeze-thaw event prior to the assessment.

### ELISA and Luminex Multiplex Array

Synovial fluid aliquots were thawed on ice and 20 μl of fluid was diluted 1:5 with the Milliplex running buffer (Millipore, Billercia, MD). Sample analysis was performed by Eve Technologies (University of Calgary) using the Fluorokine MAP Multiplex Human MMP Panel (R&D Systems, Minneapolis, MN) and the Luminex 100 platform (Luminex Corp., Austin, TX), according to the manufacture’s instruction, as well as previously published methods [26]. All samples were prepared and analyzed according to the manufacturer’s instructions included with the kits. All samples were assessed at least in duplicate and prepared standards were included in all runs. Briefly, MMP-specific antibodies are pre-coated onto color-coded micro-particles. These micro-particles along with standards and samples added to the plate and the immobilized antibodies bind the MMPs of interest. After washing away any unbound substances, biotinylated antibodies specific to the MMP of interest are added to each well. Following a wash to remove any unbound biotinylated antibody, Streptavidin-PE antibodies were added to each well. A final wash removed unbound Streptavidin-PE and the micro-particles were resuspended in buffer and read using the Luminex 100 analyzer. ELISA’s were preformed on normal (n = 10), early OA (n = 10), advanced OA (n = 10) and RA (n = 10) synovial fluid samples to measure the total protein amounts (pro and active) of MMP 9 and 13 using commercially available kits (Anaspec, Freemont CA) according to the manufactures instructions. Each sample was assayed in duplicate and the concentration was extrapolated from the included standards.

### Statistical Analysis

Statistical analysis was facilitated by Stata 9.2 for Macintosh (Stata, College Station, TX) and Prism GraphPad 5 for Macintosh (Prism, La Jolla, CA). Treatment group comparisons for MMPs and TIMPs of normal (n = 25), early OA (n = 12), OA (n = 17), and RA (n = 15), were made using the Kruskal-Wallis test with Dunn’s multiple comparisons test. Principal component analysis (PCA) was executed on all arthritic patients (early OA, OA and RA) and normal control samples, a total of 69 records within the data set in which factor loadings were calculated for MMP-1, 2, 3, 7, 8, 9, 12, and 13. Principal components were retained if their Eigen value was greater than or equal to 1 (in this case 3 components were retained). To mathematically verify the visual groupings of patients of each of the four treatment groups presented by PCA, K-Means clustering was implemented using the first three principal components as inputs. K – Means clustering is an unguided algorithm that separates data into a predetermined number of groups (k groups). This is accomplished through an iterative process that calculates the shortest distance in space between 3 randomly selected centoids, then allocates each sample accordingly. The distances are calculated based on the multivariate data that can be associated with each sample. In our study, each sample was represented by three data points, the first, second, and third principal components. PCA is a data reduction algorithm that produces components that represent ideally weighted values that are calculated from the contributions of all MMPs investigated, while retaining much of the variability of the data set.

## Results

### Group Comparison

**Figure 1:**
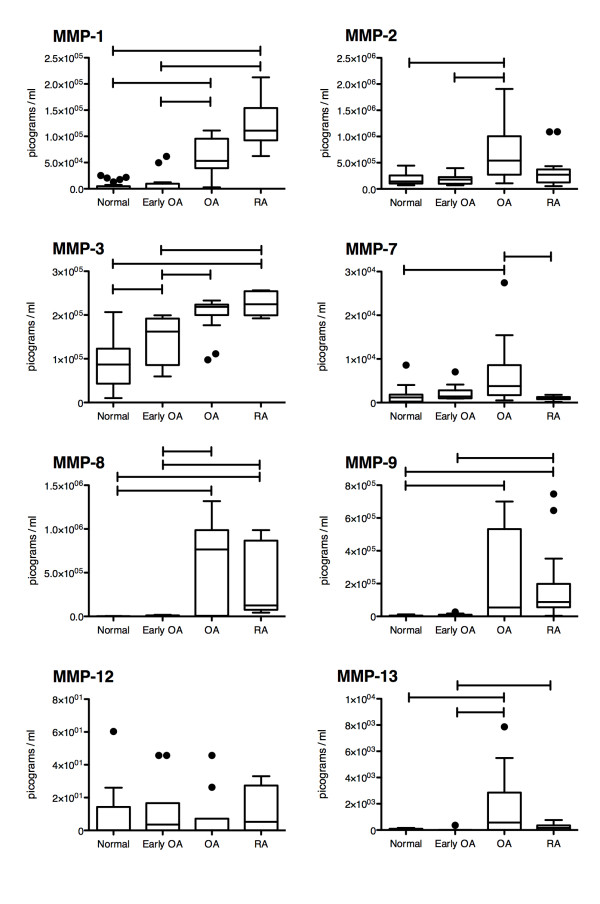
** Comparison of MMP protein expression within the synovial fluid of Normal (n = 25), Early OA (n = 12), advanced OA (OA) (n = 17), and RA (n = 15) patients.** Significant differences indicated by bars connecting treatment groups. Box and whisker plot using Tukey’s method, individual dots represent outliers. Significance accepted at p < 0.05.

**Figure 2:**
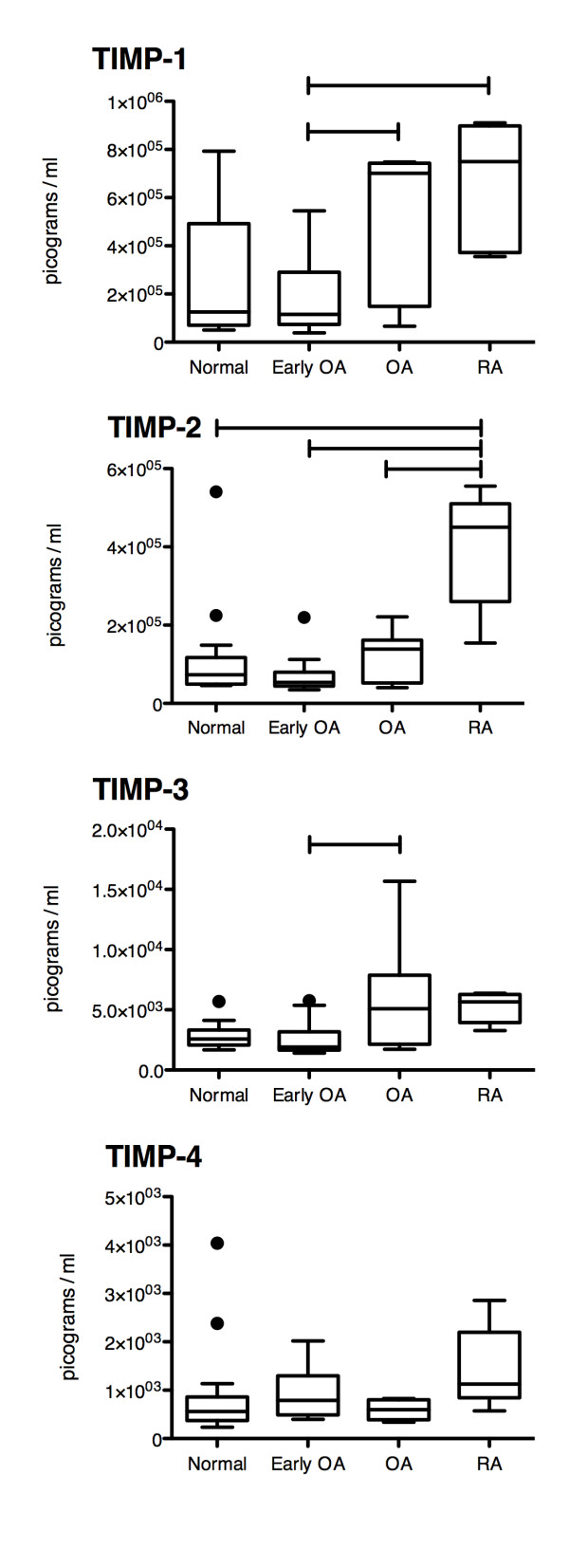
** Comparison of TIMP protein expression within the synovial fluid of Normal (n = 25), Early OA (n = 12), advanced OA (OA) (n = 17), and RA (n = 15) patients.** Significant differences indicated by bars connecting treatment groups. Box and whisker plot using Tukey’s method, individual dots represent outliers. Significance accepted at p < 0.05.

**Figure 3:**
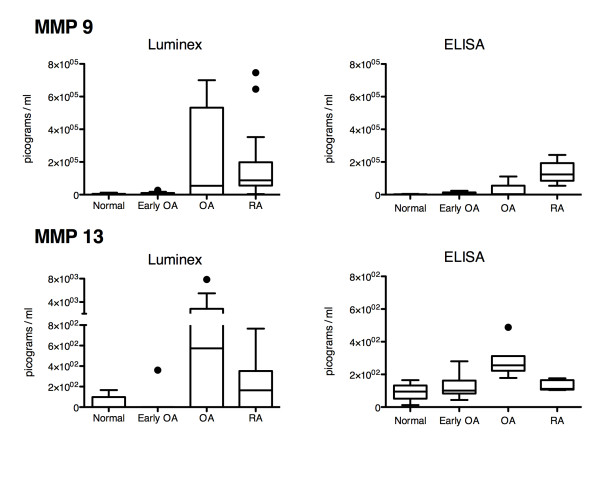
** Qualitative comparison of MMP 9 and 13 protein expression levels returned by Luminex multiplexing and ELISA.** Box and whisker plot using Tukey’s method, individual dots represent outliers.

Multiplex analysis was undertaken on synovial fluid samples from normal, early OA, OA and RA joints to investigate protein expression levels of MMPs (Figure 1) and TIMPs (Figure 2). Investigation of MMP protein expression levels (Figure 1) revealed a significant difference in MMP 3 when normal samples were compared to early OA samples. MMP 1, 2, 3, 8, and 13 expression levels were significantly elevated in advanced OA samples when compared to early OA samples, while also significantly elevated when compared to MMP 1, 2, 7, 8, 9, and 13 expression levels of normal synovial fluid samples. When RA MMP levels were compared to normal MMP synovial fluid levels, significant elevations of MMP 1, 3, and 8 were observed within the RA samples. Finally, MMP 7 expression was significantly decreased in RA samples compared to OA samples. Since multiplex analysis is a relatively new methodology and a number of studies have validated the accuracy of the approach, some discrepancies have also been observed between multiplex assays and ELISA [26-30]. Therefore, analysis was conducted on MMP 9 and 13 to verify the trend of expression levels across treatment groups as reported by Luminex multiplexing to those detected by ELISA. With the exception of Luminex being more sensitive to MMP 9 & 13 in advanced OA synovial fluid (although the medians are similar, black bars within each treatment group), the expression trends (and medians) appear the same in each of the quantification methodologies (Figure 3).

Since the assays (both multiplex and ELISA) detected total MMP levels (active and pro forms) it was decided to assay for the levels of TIMPs (tissue inhibitors of metalloproteinases) in all synovial fluid samples to determine the expression profile of MMP inhibitors present under normal and arthritic conditions. Investigation of TIMP protein expression levels (Figure 2) revealed a significant increase in expression level when RA samples were compared to normal samples (TIMP 2), early OA samples (TIMP 1, 2), and advanced OA samples (TIMP 2). Advanced OA sample expression levels were elevated when compared to early OA samples for TIMP 1, and 3. Although a number of significant differences in MMP protein expression were observed between normal, early OA, advanced OA and RA synovial fluid, variation was observed within the MMP expression panels of subjects within the four groups. Therefore principle component analysis was used to determine how MMP levels could be weighted towards specific joint disease states.

### Principal Component Analysis

**Figure 4:**
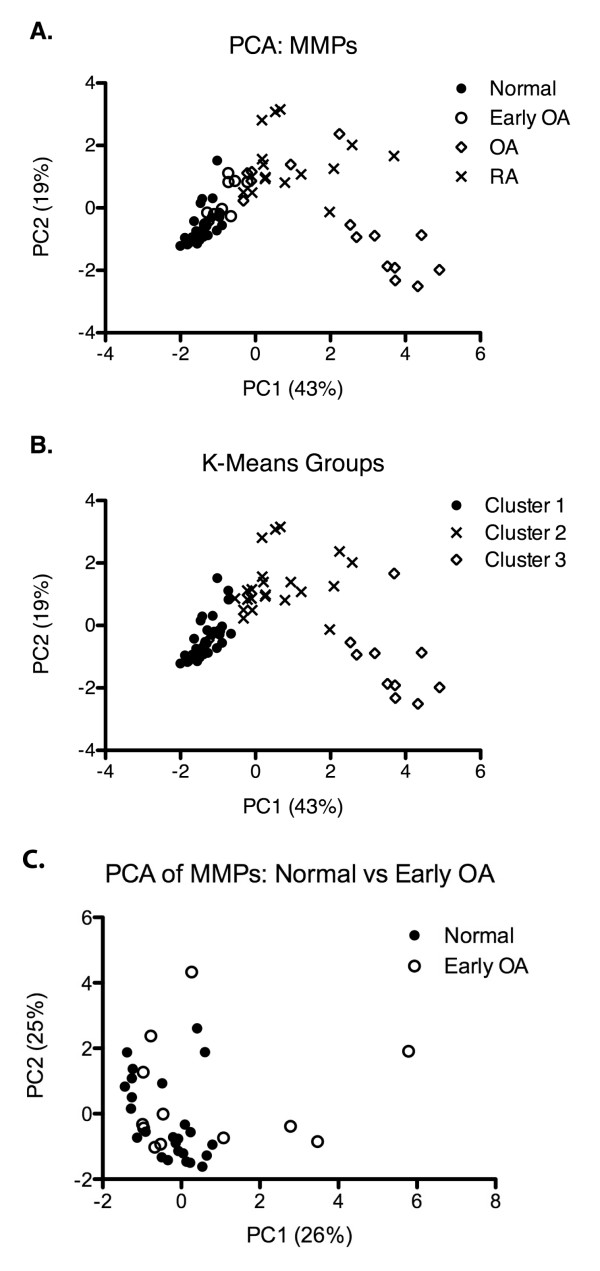
**(A.) Score plot of Principal component analysis on MMP protein expression within the synovial fluid of all samples collected (n = 69).** 3 groupings of samples are visible along the axis of component 1, normal control (CTRL) and early OA patients appear grouped together while RA and advanced OA (OA) patients appear to form separate groups. Along the axis of component 2 there is a less distinct separation of treatment groups, however RA and OA appear to be separated with overlap form the normal and early OA. (**B**.) K-Means clusters to verify the 3 potential groupings of patients. Cluster 1 contains 25 normal, 10 early OA, and 2 OA patients. Cluster 2 contains 14 RA, 2 early OA, and 6 OA patients. Finally, Cluster 3 contains 9 OA and 1 RA patients. (**C**.) Score plot of Principal component analysis on MMP protein expression within the synovial fluid of only normal and early OA samples.

PCA was performed on the quantified MMP concentration data obtained from the multiplex analysis. It was found that our data set of 8 MMPs could be collapsed into 3 principal components that maintained 78% of the total variance of the data set (Table 2.A). Three visual groupings of our patient data were apparent upon interpretation of the score plot (Figure 4A.) of the first two principal components (PC1, and PC2). These three groups appear to represent a combination of normal and early OA patients, as well as groups of OA and RA patients separately. PC1 and PC2, represent 43% and 19% of data set variance respectively, additively accounting for 62% of the variation within the data set. The factor weights, or the loading of each MMP on PC1 and PC2 (Table 2.A) suggest that MMP 2, 8, and 9 were the most influential in separating arthritis samples (both OA and RA) from each other, as well as from normal and early OA synovial fluid samples, while MMP 1, 12, and 13 where the most influential in the vertical separation of RA synovial fluid samples from OA samples. Through the use of this statistical method, specific MMP profiles/weights can be attributed to normal, early OA, OA and RA synovial fluid samples. However, to verify the visual groupings apparent in the score plot a clustering algorithm was used to test if these profiles could be validated to predict the joints state solely on the MMP expression levels.

**Table 2:** Summary of Principle Components Analysis, Components and Factor Weights for normal, early OA, advanced OA (OA) and RA

**A. PCA of Normal, Early OA, OA, and RA**				
PC1	3.44	43%		
PC2	1.53	19%		
PC3	1.09	14%		
PC4	0.76	10		
**MMP**	**PC1**	**PC2**	**PC3**	
MMP	0.2704	0.5714	-o.2679	
MMP2	0.4037	-0.2209	-0.2829	
MMP3	0.3665	0.416	-0.2448	
MMP 7	0.2875	-0.1492	0.6697	
MMP8	0.4962	-0.1641	-0.0277	
MMP9	0.4130	0.0671	0.4098	
MMP12	0.0239	0.5104	0.3486	
MMP13	0.3599	-0.3706	-0.2225	
**B. PCA of Normal and Early OA**				
Component Eigenvalue Proportion				
PC1	20.7	26%		
PC2	1.97	25%		
PC3	1.51	19%		
PC4	1.09	14%		
PC5	0.49	6%		
**MMP**	**PC1**	**PC2**	**PC3**	**PC4**
MMP1	-0.2668	0.5118	0.2046	0.2665
MMP2	-0.3357	0.5045	0.0452	0.0662
MMP3	0.1353	0.1077	0.6581	0.3545
MMP7	0.1996	0.4664	-0.1648	-.04650
MMP8	0.4399	0.2325	-0.2258	0.5606
MMP9	0.5954	0.0967	-0.2546	0.1539
MMP12	0.3143	0.3696	0.2386	-0.4688
MMP13	0.334	-0.2333	0.5685	-0.1580

### Clustering Analysis of MMP Expression Levels

Since distinct MMP expression profiles were observed in normal, OA and RA synovial fluid from the PCA (Figure 4A), these profiles were analyzed using a clustering algorithm to test the specificity of each MMP profile (Figure 4B). Using PC1, PC2, and PC3 as ideally weighted inputs, the K-means clustering algorithm was set to look for 3 distinct clusters. This algorithm returned a normal/early OA cluster (25 normal, 10 early OA, and 2 advanced OA), a RA cluster (14 RA, 2 early OA, and 6 advanced OA), and an advanced OA cluster (advanced 9 OA, and 1 RA) (Figure 4B.). Thus, mathematically verifying the visual grouping of data by PCA, and suggesting that distinct MMP expression profiles reside in normal / early OA, advanced OA, and RA knee joints across a wide range of age and prescribed medication, with contributions from both males and females. Since the normal and early OA groups appear to co-localize on the PCA and clustering plots, a further validation/verification step was taken to confirm this result. When examining the normal and early OA data separately using PCA analysis, it was made clear that the normal and early OA synovial fluid MMP expression profiles are indistinguishable from each other (Figure 4C, loading values presented in Table 2.B).

## Discussion

In this study, the levels of select MMPs and TIMPs were compared in the synovial fluid of normal, early OA, advanced OA and RA afflicted joints. It was demonstrated using multiplex Luminex technology and PCA that distinct MMP profiles are present in advanced OA and RA samples. Previous studies have demonstrated MMP 1, 3, and 9 are elevated in the synovial fluid of RA patients compared to levels present in advanced OA patients [15], however, conflicts in the comparison of our results to previous studies need to be addressed. A comparison to similar, previously published studies has been provided in Table 3. Table 3 summarizes our results, and then compares to the results of similar studies conducted by Kim et. al. [31], Yoshihara et. al. [15], Matsuno et. al. [32], and Liu et. al. [17], that have also investigated MMP profiles of late-stage OA and RA. Although these studies did not investigate MMP expression in normal or early OA synovial fluid, they do provide a valuable comparison between RA and OA expression profiles. Differences between our findings, and those of others, have been highlighted in bold. We detected a non-significant decrease of MMP 2 expression in RA synovial fluid compared to advanced OA synovial fluid, while Kim et al. [31] and Yoshihara et al. [15] each reported increases. We were able to detect a significant decrease of MMP 7 expression levels in RA samples compared to advanced OA samples, however, no differences in MMP 7 expression were detected in a study by Matsuno et. al. [32]. In contradiction to Yoshihara et al., [15] our findings indicate that a trend found within MMP 8 of RA synovial fluid samples was less than what was present in advanced OA synovial fluid. Finally, our investigation of MMP 13 revealed a decrease in expression level within RA synovial fluid compared to advanced OA, although not significant, a finding opposite to that of Kim et al [31]. It is possible that we were not able to report differences between RA and advanced OA synovial fluid in MMP 3 and MMP 9 due to variation stemming from sample sizes and patient selection. Furthermore, it is quite possible that patients with differing levels of disease severity and/or activity may yield conflicting results. A study by Maeda et al., demonstrated that synovial fluid MMP 1 levels correlated strongly with joint inflammation in RA patients [33]. In the current study, we have not quantified inflammatory mediators in the synovial fluid, however, these studies are ongoing within our lab. The equal possibility exists, however, that the differences in the methodology employed in our study compared to others may have had an impact on the resulting data. The vast majority of research into MMP protein levels synovial fluid and serum/plasma uses ELISA, a method that can detect small amount of soluble protein in suspension [15,17,31,32]. Furthermore, when MMP 9 and 13 were analyzed using ELISA we observed that the ELISA detected higher amounts of each protein. It is important to note that the R&D (Luminex) and Anaspec (ELISA) kits do not utilize the same antibodies, however, at the time of publication R&D did not supply ELISA kits for MMP 9 & 13 that detect total protein. Based on our comparison of Luminex to ELISA with MMP 9 & 13, it appears that normal, early OA and RA quantification was similar, however, in advanced OA there seemed to be detected at a higher level using the R&D assay kit, yet the median values were nearly identical for each treatment group when examined with Luminex or ELISA. Although ELISA is the ‘gold standard’, it still exhibits certain limitations. For example, since most commercially available ELISA kits using horse radish-peroxidise (HRP) or alkaline phosphatase (AP) based detection, each specific antibody is required to have its own well and sample aliquot, which when assaying small volumes of sample can lead to increased well to well variability. The Luminex platform is capable of measuring the expression of 100 specific antibodies per well, resulting in decreased signal artifacts per well, in addition to increased specificity since two specific capture antibodies per molecules are required to bind before quantification takes place. These properties present Luminex as an ideal system for quantifying very small changes (pico-gram levels) in protein levels in equally small sample volumes (micro-litre levels) in a high throughput manner. Furthermore, the collection, storage and treatment of synovial fluid were also variable between our studies and those of others. Within the current study all synovial fluid samples were aliquoted and frozen once at -80^0^ C, and no enzyme (hyaluronidase) treatment was performed on the synovial fluid as we have observed this can increase sample to sample variability based on the age, time and freeze thaw number of the enzyme (data not shown).

**Table 3:** Summary of RA and advanced OA (OA) Results and Comparison to Current Literature

**MMP**	**Comparison**	**Our Result**	**Current Literature**	**References**
1	RA vs OA	+	+	Kim et. al (2011), Yoshihara et.al. (2000), matsuno et. al. (2001)
2	RA vs OA	-	+	Kim et. al (2011), Yoshihara et. al. (2000)
3	RA vs OA	~	+	Yoshihara et. al. (2000), Matsuno et.al. (2001)
7	RA vs OA	-*	ND	Matsuno et.al. (2001)
8	RA vs OA	-	+	yoshihara et.al. (2000)
9	RA vs OA	-	+	Kim et. al (2001), Yoshihara et.al. (200), Matsuno et. al. (2001)
12	RA vs OA	+	+	Liu et. al. (2004)
13	RA vs OA	-	+	Kim et. al (2001),

+ = expression in RA samples is increased comppared to OA samples; - = expression in RA samples is decreased cpmpared to OA samples; ~ = no difference; ^*^ = significant difference; ND= not detectable.

Compared to MMP research in synovial fluid, few papers have examined and quantified TIMP protein expression in arthritic knee joints and compared this to TIMP expression in normal synovial fluid. Within the present study, no significant differences in TIMP expression (1, 2, 3, or 4) were detected between normal and early OA synovial fluid, which is consistent with the findings that normal and early OA synovial fluid could not be segregated based on MMP expression levels alone. Overall the published data seems to suggest that TIMP levels do not increase significantly with the severity of OA [34,35], however, TIMP 1 levels decrease within injured joints [36]. Based on results present here and previous studies it would appear that the regulation of MMPs by TIMPs may be important in the progression of disease, however, it also appears that it cannot be clearly understood solely through the quantification and/or ratio of MMPs to TIMPs.

Using the Luminex platform, MMP-1, 2, 3, 7, 8, 9, 12, 13 and TIMP-1, 2, 3, 4 synovial fluid protein levels were quantified in normal, early OA, advanced OA and RA joints. Although there may be some concern over using cadaveric donations in this case, it was necessary to completely define the joints as ‘normal’. In some studies, patients requiring arthroscopic examination that do not demonstrate obvious pathology are considered normal, while in others synovial fluid is simply removed from joints of individuals that have no outward symptoms of arthritis.

For the present study, synovial fluid was chosen as MMP expression within the joint may or may not be correlated to that of serum/plasma [37-39]. Since cartilage degeneration of the knee takes place within the synovial boundary it makes sense to sample and test the synovial fluid for up/down-regulation of MMPs in the joint environment. Although cell sources of MMPs within the joint vary, for example synoviocytes (MMP 1, 2), chondrocytes (MMP 3, 7, 13), neutrophils (MMP 8, 9) and macrophages (MMP 12), with significant redundancy in expression of each MMP by multiple cell types, the results presented in this study (on MMP expression) correspond with previous cellular studies. Mature macrophages expressing MMP 12 are equally represented in the synovial membrane and fluid of OA and RA patients [40,41], and in both disease states are significantly increased in number compared to normal synovial tissue [41], importantly though, MMP expression levels within a given cell type can also change with disease state [17]. Recently, through FACS analysis of synovial fluid of OA and RA patients it was identified that the levels of neutrophils are actually not significantly different [42]. Furthermore, past studies have demonstrated that neutrophils in RA presented diminished phagocytic activity compared to other types of arthritis [43]. Overall these results corroborate our MMP expression profiles (specifically MMP 8, 9 and 13) and suggest that although there are similar numbers of neutrophils in OA and RA, they may be expressing less MMPs in RA synovial fluid. In specific regards to MMP 2, a number of studies have clearly demonstrated that more fibroblasts are present in OA synovial fluid than RA in part validating this result [41,44].

Although using MMP profiles within synovial fluid cannot distinguish normal vs. early OA knee joints, this methodology maybe applicable to drug/treatment testing, since this approach is based on a number of markers, even if one of two are differentially regulated within an individual, the overall profile of disease will not be significantly affected depending on the number of targets used. A number of MMP inhibitors have been developed to treat arthritic diseases with little or no effect, however, using this approach the overall effect of treatment (including related and ‘off’ targets) per individual profile could be determined using this high throughput approach. This would allow researchers, physicians and pharmaceutical companies to not only examine the effect of a specific drug on a specific target, but also visualize the effects more globally among a family of targets, which could lead to more efficient treatments for arthritis.

## Conclusions

Although many studies have compared synovial fluid expression levels or protein to those of serum samples to look for corresponding targets to be exploited as biomarker profiles, it may be unlikely that only one or two proteins demonstrate reliable specificity with OA or RA. Using the methodology outlined in this study, the relationship between expression levels of MMPs can be merged into a single visual plot which allows for the reliable and reproducible identification of normal from diseased joints, however, this methodology cannot distinguish between normal and early OA synovial fluid, suggesting that the ‘degradative snap shot’ of the early OA joint may not be significantly different from that of a healthily joint at the level of the MMPs analyzed.

## Abbreviations

OA, Osteoarthritis; RA, Rheumatoid Arthritis; MMP, Matrix Metalloproteinase; TIMPs, Tissue Inhibitor of Metalloproteinases; PCA, Principle Component Analysis; CP, Citrullinated Peptides; RF, Rheumatoid Factor; ECM, Extra Cellular Matrix; SAOTDP, Southern Alberta Organ and Tissue Donation Program Horse Radish; HRP, Peroxidase; AP, Alkaline Phosphatase.

## Competing interests

The authors of this manuscript have no competing interests to disclose.

## Authors’ contributions

BJH: Statistical data analysis, graphical data presentation, manuscript writing. LM: Providing patent samples, manuscript editing. JBR: Providing samples, manuscript editing. CBF: Providing patient samples, manuscript editing. DAH: Project design, manuscript editing. RK: Project creator and leader, manuscript direction and writing.

## Pre-publication history

The pre-publication history for this paper can be accessed here:

http://www.biomedcentral.com/1471-2474/13/126/prepub
